# Design of the e-Vita diabetes mellitus study: effects and use of an interactive online care platform in patients with type 2 diabetes (e-VitaDM-1/ZODIAC-40)

**DOI:** 10.1186/1472-6823-14-22

**Published:** 2014-03-04

**Authors:** Yvonne Roelofsen, Steven H Hendriks, Floor Sieverink, Michael van Vugt, Kornelis JJ van Hateren, Frank J Snoek, Maartje de Wit, Rijk OB Gans, Klaas H Groenier, Julia EWC van Gemert-Pijnen, Nanne Kleefstra, Henk JG Bilo

**Affiliations:** 1 Diabetes Centre, Isala, Zwolle, The Netherlands; 2 Center for eHealth Research and Disease Management, Department of Psychology, Health and Technology, University of Twente, Enschede, The Netherlands; 3 Department of Medical Psychology, VU University Medical Center, Amsterdam, The Netherlands; 4 Department of Internal Medicine, University of Groningen and University Medical Center Groningen, Groningen, The Netherlands; 5 Department of General Practice, University Medical Center Groningen, Groningen, The Netherlands; 6 Langerhans Medical Research Group, Zwolle, The, Netherlands

**Keywords:** Patient web-portal, Health-related quality of life, Quality of care, Type 2 diabetes mellitus, Self-management, Telehealth

## Abstract

**Background:**

Due to ongoing rise in need for care for people with chronic diseases and lagging increase in number of care providers, alternative forms of care provision and self-management support are needed. Empowering patients through an online care platform could help to improve patients’ self-management and reduce the burden on the healthcare system.

**Methods:**

Access to laboratory results and educational modules on diabetes will be offered through a platform for subjects with type 2 diabetes mellitus treated in primary care. Differences in socio-demographic and clinical characteristics between subjects expressing interest vs. disinterest to use the platform will be explored. Platform usage will be tracked and compared. Patient satisfaction and quality of life will be measured by validated questionnaires and economic analyses will be performed.

**Discussion:**

This study is designed to assess the feasibility of use of an online platform in routine primary healthcare for subjects with type 2 diabetes mellitus in the Netherlands, and to study effects of use of the platform on treatment satisfaction, quality of life and clinical parameters. Although providing access to a online platform is not a novel intervention, usage and effects have not yet been studied in this patient population.

**Trial registration:**

Trial registration: NCT01570140.

## Background

Worldwide a dramatic increase is seen in the prevalence of diabetes. In 2007, the prevalence of diabetes in The Netherlands was estimated to be 740,000, while in a conservative estimate this is expected to rise to over 1,300,000 in 2025, of which more than 90% will have type 2 diabetes mellitus (T2DM) [1,2]. At the same time, the increase in number of care providers is lagging. This inevitably will result in an actual decrease in available face-to-face time for contacts between patients and care providers. This necessitates development of alternative forms of treatment and support of patients’ needs and self-management abilities beyond current situation and possibilities [2].

One possibility for reducing workload is increasing patients’ participation in their own care by promoting knowledge regarding their chronic disease(s) and insights in their own situation. Improving online access to reliable information through promoting use of an online platform may help to support such goals [3], but may also result in reducing workload for caregivers. Furthermore, by creating patient profiles, more specifically tailored educational and training can be offered. Personal contact through internet between patients and care providers will also add value. Access to credible health information and personal health data may potentially lead to better disease management and improvement of health. Also, collaborative disease tracking may decrease communication barriers between patients and caregivers. This may lead, in turn, to better understanding, more self reliance and promoting taking responsibility [3], with hopefully and eventually lower costs.

Large programs have been started and results have been analysed in the past decades (resulting in decidedly mixed results), such as the Whole System Demonstrator (WSD), to study effects of telehealth and telecare [4] and a study by Kennedy et al. on implementation of self-management support [5]. However, to see some of the positive study results translated into (cost) effective use of e-Health in routine general practice seems to be quite a challenge up to now [6]. In an effort to further explore opportunities and barriers, the current study aims to provide data access and education through an interactive care platform to T2DM patients in primary care in The Netherlands.

The use of this care platform will be offered as part of the e-Vita research program, in which a care platform will be implemented and studies in various regions of The Netherlands. Six sub-studies will be performed, three of which are in patients with different chronic illnesses: T2DM, chronic obstructive pulmonary disease (COPD) and/or chronic heart failure (CHF). The fourth study concentrates on cost-effectiveness of these interventions and will be performed by the Julius Center in Utrecht. The fifth study will assess determinants of platform usage, by using methods developed by the Center for eHealth Research and Disease Management, Department of Psychology, Health and Technology, University of Twente, Enschede. The sixth study is part of the study in T2DM patients and is performed by the Department of Medical Psychology, VU University Medical Center, Amsterdam.

The presented study is performed in T2DM patients in the Netherlands, treated in primary care. Patient empowerment is strived for by offering the possibility to track laboratory results based on the yearly check-ups, as well as offering educational modules to start a self-chosen process of lifestyle intervention through the platform. Even small improvements in lifestyle may on longer term result in moderate to large changes in self-reported dietary and physical activity behaviours that can result in (cost-effective) improvements in health related measurements (adiposity, blood pressure and lipid levels) [7,8]. Other results may be improvements in patients’ health related quality of life (HRQoL) [9], which in itself is an independent predictor of mortality in patients with T2DM [10,11]. The earlier mentioned study performed by the VU University Medical Center is a randomized controlled trial nested within the T2DM study, comparing effects of online coaching vs. no coaching of a self-management support module on self-reliance [12].

### Objectives e-VitaDM

This study will investigate the hypothesis that use of a care platform and its educational content by T2DM patients will positively influence perceived quality of life, wellbeing and degree of self-reliance [13-16]. Also, patients’ opinion regarding received care will be assessed [17,18]. Furthermore, the influence of platform use on clinical parameters, like glycaemic control, will be investigated. Besides direct benefits for patients themselves, e-health facilities could result in reduction of medical care utilization, less workload for care providers and decrease of costs of care.

### Objectives ZODIAC

All patients in the study will also be assessed in a long-term follow-up study for at least the next ten years. This prospective observational study should be considered the continuation of the Zwolle Outpatient Diabetes Project Integrating Available Care (ZODIAC) started in 1997 [19-25]. It will assess the effects of clinical parameters and quality of life on micro- and macrovascular complications and mortality. In addition, it will address relevant questions in relation to present routine of diabetes care by adding more up-to-date data in comparison with the initial phase of the ZODIAC project.

## Methods

### Study design

**Figure 1:**
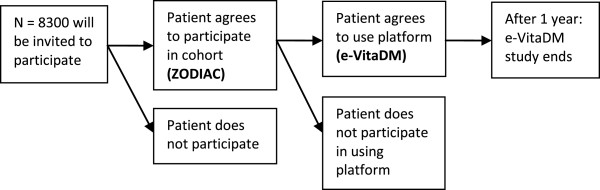
Patient flow.

**Figure 2:**
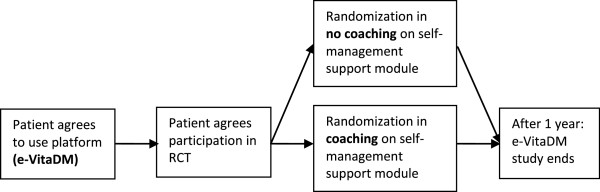
RCT nested in e-VitaDM study.

A prospective observational study of patients volunteering to use the interactive care platform (e-VitaDM), within the ZODIAC study (see Figure 1). Nested in this observational study a randomized controlled trial is planned testing coaching vs. non-coaching of a self-management support module (see Figure 2) [12].

### Study population

The included sample consists of patients with T2DM treated in primary health care in the Drenthe-region of The Netherlands as part of the shared care initiative of the Care Group Drenthe (CGD). Some specific inclusion- and exclusion criteria have been formulated:

#### Inclusion criteria

Patients can participate when they are diagnosed with T2DM (ICPC T90.2), with the GP defined as main care provider, when they are part of the Drenthe shared care initiative, and when they are aged ≥18 years.

#### Exclusion criteria

There are no exclusion criteria for the cohort study (ZODIAC). For the intervention study (e-VitaDM) exclusion criteria are:

Not participating in the shared care initiative

Mental retardation or psychiatric treatment for schizophrenia, organic mental disorder or bipolar disorder currently or in the past

Insufficient knowledge of Dutch language to understand the requirements of the study and/or the questions posed in the questionnaires

Life expectancy <1 year due to malignancies or other terminal illnesses

Cognitive impairment, including dementia, that interferes with trial participation

Any condition that the investigator of coordinating investigator feels would interfere with trial participation or evaluation of results

### Setting

The CGD is one of the care groups connected to our Diabetes Centre for benchmarking and study purposes for years. It is the largest care group in The Netherlands, including over 500,000 subjects and over 200 GP’s, divided over 110 practices. 52 out of the 110 general practices agreed to participate in the e-VitaDM/ZODIAC study. In these practices approximately 8300 T2DM patients were treated in 2011.

In line with clinical guidelines, patients in CGD are seen four times a year, of which one visit is the annual check-up. All patients with T2DM fulfilling the inclusion criteria, will be asked to participate in the prospective observational cohort study (ZODIAC) three months before their annual check-up. They are also informed about the possibility to use the care platform (e-VitaDM). Patients who express interest in using the platform are registered on the platform by their primary care nurse (PN) and will be included in the e-VitaDM study. The PN’s are trained to offer their T2DM patients information and guidance with the use of the platform and its educational content. Participants will be able to use the platform after their written consent and registration by their PN.

### Study duration

**Figure 3:**
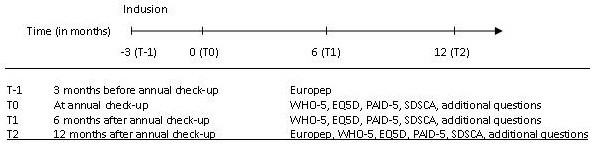
Individual timeline.

The inclusion period will last one year and the follow-up period for the e-VitaDM part will last one year and three months. This will result in a study duration of two years and three months. A complete set of data of an individual person (clinical measures and questionnaires) is available at the annual check-up and at one year. Figure 3 shows the individual timeline.

### Information on interactive care platform

A selection of DM-related health measurements as performed at the annual check-up will be provided on the platform to the individual participant, accompanied with an explanation of each item. These measurements are available from 2009 onwards, so patients will see their results over some years. Results will be displayed textually as well as graphically, showing individual values as well as the development of values over time (see Additional file 1).

Furthermore, educational information will be presented to the participant based on gender, age and actual laboratory results and clinically important measurements (e.g. HbA1c and blood pressure), using pre-programmed algorithms. In this way, a patient specific profile is created to allow a more tailored approach in offering education. After presentation of specific information to the patient, understanding of this information will be tested by a few questions. If patients allow, their care provider will receive an automated message in the electronic patient record, indicating that the participant has completed an educational module. This information can be used as input during the next visit with the GP or PN.

In addition, participants can improve their health by setting goals and actions as part of the self-management module. The development of the self-management module in e-Vita is based on the Health Action Process Approach (HAPA) model of behaviour change and the Proactive Interdisciplinary Self-Management (PRISMA) course [12].

The platform is designed with emphasis on making it suitable and available for all T2DM patients. From the start, special focus groups of caregivers and caretakers are actively involved in designing and testing of this platform.

The platform is reached through an HTTPS-connection and a multi-factor authentication. Development and hosting of the platform is based on NEN-procedure 7510, ISO 27001, Security Access Layer (SAL), Personal Data Protection Act (in Dutch: Wet Bescherming Persoonsgegevens (WBP)) and National Cyber Security Centre’s ICT Security Guidelines for web applications part 1 and 2, in which a code for protection of information is determined.

### Measurements

Table 1 shows an overview and time schedule for data to be collected in the intervention study (e-VitaDM) and the observational cohort study (ZODIAC). The collection of data follows NEN-procedure 7510 and all substandards based on the ISO 27000 series. In addition, all identifiable personal information will be encrypted in accordance with the Rijndael algorithm.

**Table 1:** Overview and time schedule for data to be collected

	**Patients using platform (e-VitaDM)**				**Patients in cohort only (ZODIAC)**			
	**T-1**	**T0**	**T1**	**T2**	**T-1**	**T0**	**T1**	**T2**
Patient profile		X				X		
Use of platform			X	X				
EQ-5D, EQ-VAS, WHO-5, PAID-5,		X	X	X		X	X	X
SDSCA	X			X	X			X
Europep								
Laboratory/clinical measures		X		X		X		X
Medication care utilization		X	X	X		X	X	X
Micro- and macrovascular complications		X		X		X		X
Mortality, assessed by PN/GP				X				X
Number of encounters with care providers				X				X
Mortality, CBS, GBA				X				X

The baseline assumption is that ≥90% will participate in the long-term study (ZODIAC), as was our experience in the earlier ZODIAC-study, but that no more than 20% of the patients will express interest in the e-Vita platform (e-VitaDM).

#### Quality assessment lists

All patients with T2DM participating in the observational cohort study (including those who are not interested in the platform) are asked to fill in a range of validated questionnaires to measure perceived quality of life (EQ-5D) [13], well-being (WHO-5) [14], diabetes-distress (PAID-5) [15], diabetes self-care behaviour (SDSCA) [16] and perceived quality of received care (Europep) [17,18]. Additional questions about coffee/tea intake, smoking habits, employment and educational background are included as well. Questionnaires are filled in at different time points (see Figure 3).

#### Clinical data gathering

Besides the questionnaires, clinical measures as described in the NHG/NAD core set of parameters will be collected (see Additional file 2). These measures are already being collected by the patients’ GP and PN during their annual check-up and are routinely sent to our Diabetes Centre for benchmark en study purposes. After informed consent of included patients, clinical data will be combined with the results of the collected questionnaires to assemble a complete and anonymized dataset. Data regarding micro- and macrovascular complications and mortality will be gathered from patient records from the GP. Mortality will also be checked by using data from Central Statistical Office (in Dutch: Centraal Bureau voor Statistiek (CBS)) or Municipal Administration (in Dutch: Gemeentelijke Basisadministratie (GBA)).

#### Data on actual contacts

Data on actual amount of patient – care provider contacts will be retrieved in a subpopulation. The availability of these data greatly depends on the information system used by primary care givers. Since five different systems (each with their own strengths and limitations) are used in the Drenthe-region, one specific information system (Medicom, which is used by 8 general practices) is chosen for detailed analysis on this subject. This system is not only known for its reliability of structured data entry, data transport and medication information, but especially for its reliable registration of number of encounters with care providers and number of hospitalizations.

#### Further sample collection and storage

In addition to the blood drawn as part of the annual check-up, two extra tubes of blood (10 ml serum and 10 ml plasma) will be collected in consenting subjects and frozen for future research on novel biomarkers.

#### Assessment of determinants of platform use

In order to find out how the platform is used in practice, log-data will be used to track individual use of the platform over time (number of log-ons, time spent per session and used elements). These log-files will be used for identifying user profiles and they will provide insight about adherence to the platform, usage patterns and what elements of the platform are used. This information provides insight in how the platform (both content and system) matches with the users [26]. In addition, specific questionnaires will be sent to all patients who expressed their interest in using the e-Vita platform, irrespective of actual use. These questionnaires include TIPI [27], PAM-13 [28], short version of PII [29], questions about trust [30], questions about general use of the Internet and questions about use of and satisfaction with the platform [31].

Furthermore, care providers will be interviewed in a semi-structured way to assess possible care provider related factors influencing patient’s platform use.

### Primary and secondary endpoints

The quality assessment in terms of EQ-5D is the primary objective in this study. A clinically relevant difference of 0.074 in the EQ-5D index score is predefined [32]. Secondary endpoints are well-being as assessed by the WHO-5 questionnaire, diabetes-distress as assessed by the PAID-5, self reliance as assessed by the SDSCA, quality of delivered care as assessed by the Europep and clinical parameters.

### Statistical analyses

#### Analyses in intervention study (e-VitaDM)

To evaluate differences in target variables over time and within groups, we will use the linear mixed model for repeated measures. Variables measured after 6 and after 12 months will be used as within-subjects variables. Baseline variables will be used as covariates. Making use of the platform or not will be used as a between-subjects factor. In case of differences between groups on relevant variables, these variables will be added as a covariate in the multivariable analyses.

#### Additional analyses in larger prospective cohort study (ZODIAC-40)

Cox proportional hazard models will be used to investigate the association between (bio)marker levels and future events (microvascular complications, macrovascular complications and mortality) with adjustment for selected confounders.

Harrell’s C statistic, a rank based measure will be used to compare how well the presence of the marker in the different models used, predicts the outcome of interest.

The need for additional analyses, i.e. measures of discrimination improvement (integrated discrimination improvement, IDI, and net reclassification improvement, NRI) will be decided for each marker separately.

### Medical ethical committee

The current study has been reviewed and approved by the Medical Ethical Committee of Isala.

## Discussion

Offering T2DM patients information, education and insight in health data through internet is not new at all. However, to our knowledge, in The Netherlands there has never been a study of the proposed scale towards feasibility, efficiency and the effects of an interactive care platform as proposed in the e-Vita research program. Furthermore, part of the assessment includes structural measurements of perceived quality of life and care.

At the start of e-VitaDM study the actual content of the care platform is limited to insight in DM-related health data of an individual patient and education. In this ongoing study, new modules will be added especially concentrating on educational content. The development of further educational content is crucial, not only to further enhance active use of the platform, but also to assess the effects of new additions and improvements in a continuous cycle using focus groups in each step. The creation of content, including 2-way communication, e-contact and e-consult, during this study and keeping track of its fit with patients’ needs is not the primary focus in this research, but nevertheless has to be taken into account, not only in striving for the largest possible effect, but also in analyzing the collected data. At the moment, communication between PN’s and patients is limited to automated messages in the electronic patient record when patients allow this in case of completing educational modules. The platform does not provide an extensive 2-way communication tool (e.g. exchange messages) on top of the automated messages, but developments concerning this are already ongoing.

A previous study by the British National Health System proves that unless personal electronic health records align closely with people’s attitude, self management practices, identified information needs and the wider care package (including organizational routines and incentive structures for clinicians), the risk that they will be abandoned or not adopted at all is substantial [33].

The WSD shows that telehealth is associated with lower mortality and emergency admission rates [34], but also that telehealth did not improve quality of life or psychological outcomes for patients with COPD, T2DM or CHF over 12 months [35]. In terms of cost-effectiveness, the WSD shows that the QALY gain by patients using telehealth in addition to usual care was similar to that by patients receiving usual care only, and total costs associated with telehealth were higher [6]. However, generalizability of the WSD-study results in terms of diagnosis, relative risks and outcomes to the Dutch situation in uncertain.

## Abbreviations

CBS: Central Statistical Office (in Dutch: Centraal Bureau voor de Statistiek); CGD: Care Group Drenthe; CHF: Chronic heart failure; COPD: Chronic obstructive pulmonary disease; EQ-5D: EuroQol – 5 dimensions; GBA: Municipal Administration (in Dutch: Gemeentelijke Basisadministratie); GP: General Practitioner; HRQoL: Health related quality of life; ICPC: international classification of primary care; LSD: Large scale demonstrator; NAD: National action program Diabetes (in Dutch: Nationaal Actieprogramma Diabetes); NHG: Dutch GP Society (in Dutch: Nederlandse Huisartsen Genootschap); PAID-5: Problem areas in diabetes – 5 questions; PAM-13: Patient activation measure – 13 questions; PII: Personal involvement Inventory; PN: Primary care nurse; QALY: Quality adjusted life years; SAL: Security access layer; SDSCA: Summary of diabetes self-care activities; T2DM: Type 2 diabetes mellitus; TIPI: Ten-item personality inventory; WBP: Personal Data Protection Act (In Dutch: Wet Bescherming Persoonsgegevens; WHO-5: WHO – Five item measure of well being; WSD: Whole system demonstrator; ZODIAC: Zwolle outpatient diabetes project integrating available care.

## Competing interests

This study has been funded by the foundation Health Within Reach (in Dutch: stichting Zorg Binnen Bereik). The foundation has no role in designing the study, data collection, data analysis, reporting the results, writing or publication of this manuscript. All authors declare that they have no competing interests.

## Authors’ contribution

YR is one of the researchers in this project and mainly drafted this manuscript. SH is the other researcher and helped to draft this manuscript. FS is the researcher for the assessment of determinants of platform use and she helped to draft this manuscript. MVV is the researcher for the randomized controlled trial on patient self-management and patient coaching and helped to draft this manuscript. KJJVH and ROBG participated in the design of the study protocol. KHG participated in the proposed statistical analyses. FJS, MDW and JEWCVGP designed part of the studies and helped to draft this manuscript. NK and HJGB conceived the principal study and participated in its design and coordination and helped to draft the manuscript. All authors read and approved the final manuscript.

## Pre-publication history

The pre-publication history for this paper can be accessed here:

http://www.biomedcentral.com/1472-6823/14/22/prepub

## Supplementary Material

Additional file 1 Clinical measurements provided on the platform are the following, as also can be seen in the figure below: HbA1c, blood pressure, lipid profile, Cockcroft, alb/kreat ratio, smoking, length, weigth and BodyMass Index (BMI). Click here for file

Additional file 2 **Diabetes Core Set.** The core set describes a minimum set of parameters, which should be digitally registered by care providers for the purpose of providing care for T2DM patients. These data are used by care providers and patients in the communication and they serve to a large extent as a basis for the calculation of indicators. The table shows a summary of the data collected for this study, a detailed description can be found at http://www.actieprogrammadiabetes.nl/images/stories/downloads/ondersteuningsaanbod/e-Diabetes_Mellitus_kernset_samenvatting.pdf. Click here for file
