# Supplementary material for: Design of the e-Vita diabetes mellitus study: effects and use of an interactive online care platform in patients with type 2 diabetes (e-VitaDM-1/ZODIAC-40)
Source: BMC Endocr Disord. 2014 Mar 4;14:22. doi: 10.1186/1472-6823-14-22 (PMC4016215; doi:10.1186/1472-6823-14-22)
Supplement: Additional file 1 — Clinical measurements provided on the platform are the following, as also can be seen in the figure below: HbA1c, blood pressure, lipid profile, Cockcroft, alb/kreat ratio, smoking, length, weigth and BodyMass Index (BMI). [file 1472-6823-14-22-S1.pdf]

Additional file 1

Clinical measurements provided on the platform are the following, as also can be seen in the figure below: HbA1c, blood pressure, lipid profile, Cockcroft, alb/kreat ratio, smoking, length, weigth and BodyMass Index (BMI).

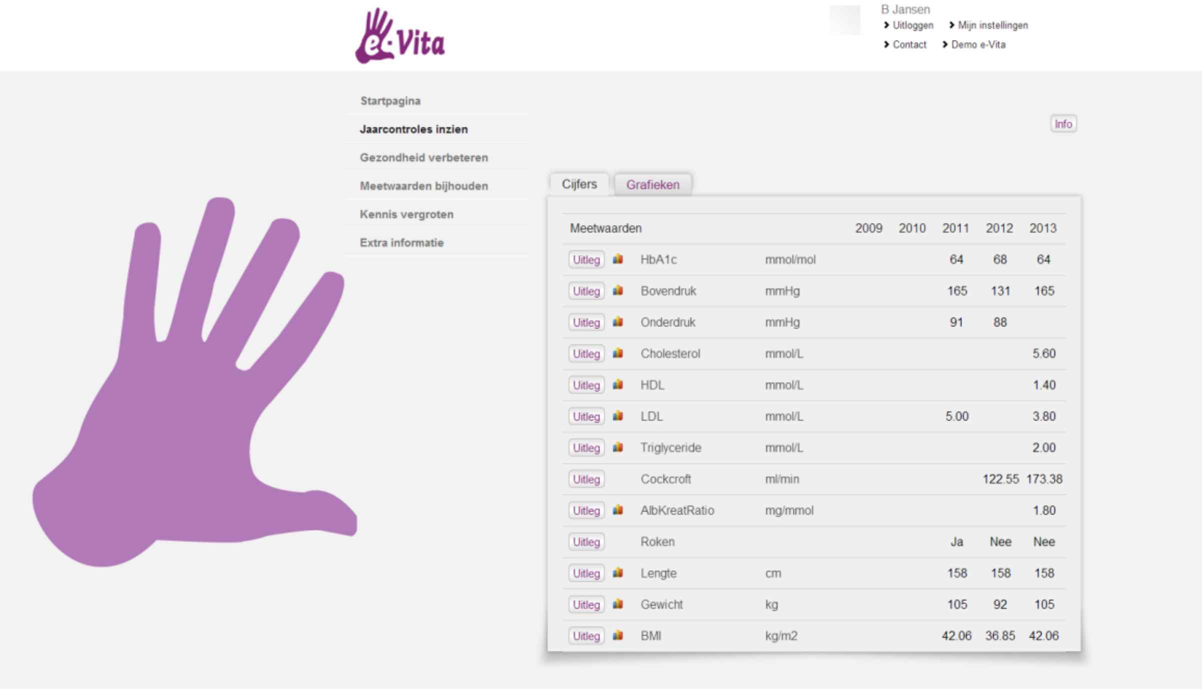

	Meetwaarden		2009	2010	2011	2012	2013
Uitleg	HbA1c	mmol/mol			64	68	64
Uitleg	Bovendruk	mmHg			165	131	165
Uitleg	Onderdruk	mmHg			91	88	
Uitleg	Cholesterol	mmol/L					5.60
Uitleg	HDL	mmol/L					1.40
Uitleg	LDL	mmol/L			5.00		3.80
Uitleg	Triglyceride	mmol/L					2.00
Uitleg	Cockcroft	ml/min				122.55	173.38
Uitleg	AlbKreatRatio	mg/mmol					1.80
Uitleg	Roken				Ja	Nee	Nee
Uitleg	Lengte	cm			158	158	158
Uitleg	Gewicht	kg			105	92	105
Uitleg	BMI	kg/m2			42.06	36.85	42.06
