# Supplementary material for: Design of the e-Vita diabetes mellitus study: effects and use of an interactive online care platform in patients with type 2 diabetes (e-VitaDM-1/ZODIAC-40)
Source: BMC Endocr Disord. 2014 Mar 4;14:22. doi: 10.1186/1472-6823-14-22 (PMC4016215; doi:10.1186/1472-6823-14-22)
Supplement: Additional file 2 — Diabetes Core Set. The core set describes a minimum set of parameters, which should be digitally registered by care providers for the purpose of providing care for T2DM patients. These data are used by care providers and patients in the communication and they serve to a large extent as a basis for the calculation of indicators. The table shows a summary of the data collected for this study, a detailed description can be found at http://www.actieprogrammadiabetes.nl/images/stories/downloads/ondersteuningsaanbod/e-Diabetes_Mellitus_kernset_samenvatting.pdf. [file 1472-6823-14-22-S2.pdf]

Additional file 2

Diabetes Core Set

The core set describes a minimum set of parameters, which should be digitally registered by care providers for the purpose of providing care for T2DM patients. These data are used by care providers and patients in the communication and they serve to a large extent as a basis for the calculation of indicators. The table shows a summary of the data collected for this study, a detailed description can be found at www.diabetesfederatie.nl.

**Parameter**	**Explanation**
Anonymized unique code	To identify the patient within the study
Physical examination	
Length	Register once
Weight	Register annually
BMI	Register annually
Systolic blood pressure	Register quarterly, alternatively register patients' self-measurement
Diastolic blood pressure	Register quarterly
Systolic blood pressure (patients' self-measurement)	Register quarterly, alternative for measurement at general practice
Diastolic blood pressure (patients' self-measurement)	Register quarterly, alternative for measurement at general practice
Systolic blood pressure (average 24 hours)	Optional
Diastolic blood pressure (average 24 hours)	Optional
Laboratory results	
HbA1c (new IFCC)	Register annually, introduced since 1 April 2010
HbA1c (old, DCCT)	Register annually, expired since 1 April 2011
Creatinine	Used for calculation or estimation of renal function
Cholesterol total	Register annually
HDL	Register annually
LDL	Register annually
Albumin (micro-) urine, portion	Register annually, choose one of the methods
Albumin/creatinine ratio	
Triglycerides	Register annually
MDRD (GFR)	Register annually, choose one of the methods
Cockcroft	
Creatinine clearance	
Foot examination	
Inspection left foot	Register annually
Inspection right foot	Register annually
Blood circulation left foot	Register annually
Blood circulation right foot	Register annually
Monofilaments examination left foot	Register annually
Monofilaments examination right foot	Register annually
SIMMS classification	Register annually
Eye examination	
Date of last fundoscopy	Advice: do not use, preferably use the option 'Fundoscopy'
Fundoscopy	Register result every 2 year

Diabetic retinopathy left eye	Register conclusion of fundus photography every 2 year. In case of retinopathy, register ICPC
Diabetic retinopathy right eye	Register conclusion of fundus photography every 2 year. In case of retinopathy, register ICPC
Risk factors	
Smoking habits	Register once in case of non-smoker, register annually in case of smokers and quitted smokers
Advice quit smoking	Register annually in case of smokers
Alcohol consumption	Register annually, choose one of the methods
FiveShot questionnaire	
Medication	
Diabetes related medication	Actual use of medication
Antihypertensive drugs	Actual use of medication
Lipid lowering drugs	Actual use of medication
Influenza vaccination (ATC)	Alternative for ICPC
Influenza vaccination (ICPC)	Register in patient record, appears usually automatically
Relevant disorders	
Cardiovascular complication / risk factors (ICPC)	K74: Angina pectoris<br>K75: Acute myocardial infarction<br>K76: Other/Chronic ischemic heart diseases<br>K86: Hypertension without target damage<br>K87: Hypertension with target damage<br>K89: TIA<br>K90: CVA<br>K92.01: Intermittent claudication<br>K92.02: Aortic aneurysms
(Micro-) vascular complications	F83.01: Diabetic retinopathy<br>U99.1: Renal impairment<br>N94.02: Diabetic neuropathy
Psychiatric disorders	P03: Feeling down, depressive<br>P76: Depression
Other history	Other actual history
